# Measuring entrepreneurship in adolescents at school: New psychometric evidence on the BEPE-A

**DOI:** 10.1371/journal.pone.0250237

**Published:** 2021-04-23

**Authors:** Javier Ortuño-Sierra, Esther Gargallo Ibort, Ana Ciarreta López, Josep María Dalmau Torres

**Affiliations:** Educational Sciences Department, University of La Rioja, Logroño, La Rioja, Spain; Universita degli Studi di Milano-Bicocca, ITALY

## Abstract

The economic situation worldwide demands individuals with entrepreneurial skills and aptitudes. The adolescence stage is a critical period in which these abilities could be developed and the school is a relevant setting for this purpose. To this end, instruments that allow assessing enterprising abilities are needed. Nonetheless, there remains a lack of instruments with adequate evidence of validity. The purpose of this study was, thus, to analyze the psychometric properties of the Battery for the Assessment of the Enterprising Personality-Adaptive (BEPE-A). The sample included a total of 1105 participants (men = 528; 47.4%) with an age range from 12 to 19 years (*M =* 15.23 years; *SD* = 4.40). The BEPE-A and the Entrepreneurial Attitudes Scale for Students (EASS) were used in the study. The EFA, conducted in a subsample of 512 participants, revealed that each of the subscales of the BEPE-A were basically unidimensional. The CFA, conducted in a second subsample of 593 participants, showed that a bifactor model best fit the BEPE-A structure. In addition, measurement invariance was found both by gender and age. The BEPE-A was positively associated with other measures of entrepreneurship. Results found in the study contribute valuable information about new evidences of a battery that allows screening for entrepreneurship in a critical developmental period such as adolescence, and in a relevant setting like school.

## Introduction

Entrepreneurial activity has received and increasing amount of attention in recent decades, pointing out the necessity to assess entrepreneurship, aspirations, and attitudes of individuals across different countries [1]. It is well known that economic as well as social and demographic factors may have an important impact when starting a business, however the role of individual variables like specific personality variables has been documented as having a higher relevance [2].

Within this context emerges the idea of entrepreneurship as a person with knowledge and abilities to detect sudden changes in the market and to innovate [3]. The entrepreneur becomes, thus, in a person with a key role in the process of developing and changing society [1]. Bearing this in mind, new initiatives have emerged globally to detect and promote entrepreneurial activity [4]. Different studies have analyzed the enterprising personality, both from a broad perspective including personality traits such as the Big-Five [2], and focused on particular traits [5]. Specific traits like achievement motivation, risk taking or innovativeness among others have been identified as related to enterprising [5–7]. It has been confirmed that the study of specific traits have more predictive power when compared to broader measures like, for instance, the Big-Five. In particular, motivation, autonomy, self-efficacy, innovativeness, internal locus of control, and risk taking have revealed a strong capacity to predict enterprising behaviour [8–10]. In addition, there are studies that have analyzed the connection between enterprising and different personality variables like, among others, achievement motivation, risk taking, innovation, stress tolerance, internal locus of control, and optimism [1,6–8,11]. For instance, a recent study reveals that vocational interests was related to educational track choices, as well as achievement goals and the Big Five personality traits [12].

Previous research has shown that entrepreneurial spirit has a key role in western modern economies [13]. In addition, entrepreneurial activity seems to play a relevant role in the development of a market economy [14]. Therefore, educational policies should be devoted to detect and educate specific enterprising characteristics that could promote transformation and advancement of the economy. The school has, thus, a key role in developing entrepreneurship and competences such as knowhow and innovation, both during childhood and adolescent ages [15].

In particular, entrepreneurship education during adolescence is believed to have a big impact in enterprising attitudes during adulthood [15,16]. Adolescence is a crucial developmental stage in which different changes occur. Acquisitions and changes during adolescence could translate into permanent behavior traits in adulthood. Relevant aspects such as autonomy, self-concept, identity or vocational aspects develop and establish during this stage. Thus, educational policies should focus on evaluating entrepreneurship during adolescence and then, promoting, and implementing new strategies to enhance an aspect that seems crucial for the individual and the society [17]. Nonetheless, there is a lack of studies analyzing enterprising personality during adolescence. In particular, in Spain, only few studies have focused on this relevant aspect in adolescents [9,18]. In addition, it is worth noting that entrepreneurial activity may be different with regards to different variables such as gender or age. With this regard, different works reveal that women prefer to grow their businesses slowly and are less likely to take risk, being more conservative in assuming growth strategies [19,20]. Nonetheless, effect sizes of the mentioned studies were small. With respect to age, a progression of age was linked to higher levels of entrepreneurial activity and wisdom [21]. However, other studies indicated that energy and motivation levels were greater in younger individuals who in addition were more flexible and more prone to engage in risk-taking behaviors [21–23].

There are different instruments that can be found with the purpose to assess this. A relevant instrument for measuring enterprising personality, that has shown adequate psychometric properties, is the Entrepreneurial Attitudes Scale for Students (EASS) [24]. Also, the Entrepreneurial Aptitude Test [25] was one of the first measures to approach to general attitudes towards entrepreneurship. In addition, the Skills Confidence Inventory [26], showed adequate psychometric properties to screen for innovative abilities. With this regard, the Measure of Entrepreneurial Talents and Abilities, META [27] is one of the most used and accepted tools combining the measurement of innovative abilities and entrepreneurship. Some other instruments are the General Enterprising Tendency [28], the Entrepreneurial Intention Questionnaire [29]. More recently, the High Entrepreneurship, Leadership and Professionalism, HELP [30] has also shown adequate validity evidences for measuring these aspects.

Worth noting is the fact that the mentioned instruments focused only on particular dimensions of entrepreneurship [31,32], thus, not allowing for the establishing of a complete profile of enterprising personality. In addition, most of the instruments developed are designed for adults [29,33,34], preventing the screening of enterprising personality in adolescents [17]. Within this context, the Battery for the Assessment of the Enterprising Personality (BEPE) is an assessment tool that comprises eight dimensions of entrepreneurial personality gathered after a complete analysis of the existing instruments found in the literature. The BEPE, allows for the screening of enterprising personality in adolescents and young people. The BEPE has shown evidences of validity and reliability of the scores [7,8]. In this regard, it should be noted that a reduced version of the BEPE, with 16 items, has been recently validated [35]. The computerized adaptive version of the BEPE, the BEPE-A (BEPE-Adaptative) [18] has also revealed adequate psychometric properties with a reduced pool of items. The final pool of items of the BEPE-A was 108 compared to the total of 127 of the BEPE. Also, and despite the fact that the BEPE-A was developed to be used as an adaptive computerized version, it is our opinion that the total final pool of items, reduced compared to the BEPE, can be a good solution to assess enterprising personality in adolescents. Currently, there is a need for short, simple instruments with adequate psychometric characteristics that enable rigorous evaluation. It is well known that shorter instruments have a positive impact in adolescents, as well as clinicians and researchers, as they are less time consuming. In this sense, the BEPE-A, as a reduced, version of the BEPE could have and added value for research purposes.

However, despite the promising psychometric properties of the instrument, only few studies have analyzed its psychometric adequacy in adolescent population, and, in particular, there is only one study assessing the psychometric properties of the BEPE-A [18]. Therefore, more studies are needed with different populations (communities) and in other regions in order to gather enough validity evidences for its use with adolescents and young people. In addition, other techniques such as the measurement invariance (MI) could add value to the study of this relevant instrument. Previous studies [18] have analyzed the differential item functioning (DIF), a somehow similar technique, of the BEPE-A in adolescent populations. The evaluation of MI or DIF are important in order to assure the generalizability of latent constructs across groups [36]. Nonetheless, and to the best of our knowledge, no other studies have analyzed these relevant aspects in instruments measuring enterprising personality.

Considering all this, the main goal of the present study was to further study the psychometric properties of the Spanish version of the full pool of items of the BEPE-A battery in adolescents at school by means of paper-based-assessment. We therefore, analyzed the internal structure of the instrument and the reliability of the scores, studied the measurement invariance of the instrument, and obtained evidences about the relationship with other variables.

## Method

The Study was approved by the Ethics Committee of the University of La Rioja.

### Participants

The initial sample comprised a total of 1255 adolescents. With the aim to guarantee the representability of the sample, different cities and different types of secondary schools–public, grant-assisted private, private, and vocational/technical schools belonging to La Rioja, a region of the north of Spain, were included. Ten schools and educational centers were used. The students belonged to different socioeconomic levels. Exclusion criteria included a diagnostic of intellectual disability and language problems. Attending to the Listwise deletion method, participants with missing values were eliminated. The final sample comprised a total of 1105 participants (men = 528; 47.4%). Participants volunteered to take part in the study (convenient samples). Participants’ ages ranged from 12 to 19 years (*M =* 15.23 years; *SD* = 4.40).

### Instruments

The **BEPE-A** battery [18]. The BEPE is an instrument developed with the aim to assess eight specific personality dimensions (Self efficacy, Autonomy, Innovativeness, Internal locus of control, Achievement motivation, Optimism, Stress tolerance, and Risk taking) considered as the most relevant in order to understand entrepreneurial personality [7,8]. The BEPE-A is composed of 108 items (e.g. I like to do new things) in a Likert-type response format with five options (1 totally disagree, 5 totally agree). A detailed description of each subscale can be found in Suárez-Álvarez et al. [18]. The BEPE-A has shown adequate evidences of validity and reliability of the scores in previous studies [18]. In this study we applied the total of 108 items.

The **Entrepreneurial Attitudes Scale for Students** (EASS) [24]. The EASS is an instrument that measures enterprising personality and is composed of 18 items (e.g. I want to have everything necessary to move forward and be a pioneer in my professional field) in a Likert-type response format with 7 options. Items are grouped in 6 dimensions: proactivity, professional ethic, empathy, innovation, autonomy, and risk taking. The psychometric properties of the EASS has been studied in previous studies [37]. The reliability of the scores in the present study were between .66 and .88 for the different subscales.

### Procedure

The instruments were administered collectively, in groups of no more than 20 students and during regular school time in a classroom specially prepared for the study. First, we contacted with the schools’ directors (headmasters) and then with parents and legal tutors who provided written informed consent for students under 18 years old. All the participants that were asked agreed to participate in the study. In addition, teachers and administrative staff at the schools where the study took place provided consent before starting the research. Participants knew before hands about the confidentiality of their responses, the voluntary nature of the study, and the fact that no incentive was provided for their collaboration. Researchers also informed participants that they could leave the experiment at any moment for any reason they considered. All of the participants who took part in the study completed the study. Researchers previously trained for the study controlled and supervised the administration of the questionnaires whichwere administered on a paper-based form.

### Data analysis

The first step involved a cross-validation study was conducted in order to randomly divide the total sample into two different subsamples. Two subsamples were obtained with a total of 512 and 593 participants respectively. In the first subsample we conducted different exploratory factor analysis in each dimension of the BEPE-A. The polychoric correlation matrices were used for Factor Analyses. We used the ULS (Unweighted Least Squares) as an extraction method. With the aim to retain the number of factors, the parallel analysis, the percentage of variance explained, and the model fit indices based on study of residuals (GFI and RMSR) were used [38]. Then, in a second step, different confirmatory factor analysis (CFA) were performed in the second subsample. Based on previous studies, and the results of the EFA, three different models were studied: a model with 8 first-order factors, a model with 8 first-order factors and a second order factor, a Bifactor model with a general factor and 8 group factors. The parameters were obtained from the Muthen’s quasi-likelihood estimator. The following goodness-of-fit indices were used: Chi-square (X^2^), Confirmatory Factor Index (CFI), Tucker-Lewis Index (TLI), Root Mean Square Error of Approximation (RMSEA), and and Weighted Root Mean Square Residual (WRMR). Hu and Bentler [39] suggested that RMSEA should be .06 or less for a good model fit and CFI and TLI should be .95 or more, though any value over .90 tends to be considered acceptable and WRMR values less than .08 as a good model fit.

We then calculated descriptive statistics of each subscale and McDonald’s Omega in order to study the reliability of the BEPE-A scores.

Next, and with the aim to study the MI by gender and age, successive multigroup CFAs were conducted. With the aim to compare age, we established two different groups: younger adolescents (12–15 years old) and older adolescents (16–19 years old), attending to the initial and final stages of adolescence [40]. The study of MI is performed frequently by multigroup comparisons through structural equation modelling under the measurement models [36]. First, the configural invariance model was established with items constrained to load on the same factors across groups, but all item thresholds and factor loadings were free to vary across groups. We established a strong invariance model, which contained cross-group equality constraints on all factor loadings and item thresholds. Moreover, factor means fixed to zero in the first group and free in the other groups and scale factors fixed to one in the first group and free in the other groups. Considering the limitations of the Δχ2, the ΔCFI criterion was used to establish if nested models are practically equivalent Cheung and Rensvold [41]. Thus, if the change in CFI is less than or equal to .01, it is possible to continue with the next step in the analysis of MI.

Then, we calculated latent mean differences across gender and age. Statistical significance was based on the *z* statistic. The group in which the latent mean was fixed to zero was considered as the reference group. In addition, we computed Cohen’s *d* [42] to investigate the effect sizes of the latent mean differences. A value of *d* ≥ 0.2 was considered a small effect, a *d* ≥ 0.5 medium effect, and *d* ≥ 0.8 a large effect.

In order to obtain evidences of convergent validity, Pearson’s correlations between the BEPE and the EASS were calculated.

SPSS 15.0 [43], FACTOR 10.5.01 [44], and Mplus 7.0 [45] were used for data analyses.

## Results

### Evidences of internal structure for the BEPE

The analysis of the EFA in the first subsample revealed statistically significant values of Bartlett’s Sphericity Index with a value of 3439.7 (p < .001) and Kaiser–Meyer–Olkin (KMO) indices above 0.85 in all cases. The GFI values were above .95 in all the dimensions and the RMSR was under .08. The first factor explained more than 30% of the variance in all the dimensions. Thus, the different dimensions should be considered as unidimensional.

After the EFA was conducted, different CFA were performed at the item level. As seen in Table 1, the 8-factor solution and the factor solution with a second order factor revealed CFI and TLI values close but under the .90 cut-off. The bifactor solution depicted adequate goodness-of-fit indices, including CFI and TLI values above .90 and RMSEA under .08.

**Table 1:** Goodness-of-fit indices of the dimensional models tested.

Models	χ^2^	*df*	CFI	TLI	RMSEA (90% C.I.)	WRMR
8- single factors	1740.57	153	.82	.85	.11 (.11-.12)	2.30
8 single factors plus a second order factor	874.41	168	.88	.87	.06 (.06-.07)	1.99
Bifactor model with 8 factors	576.99	148	.92	.91	.05 (.05-.06)	1.32

*Note*. χ^2^ = Chi square; *df* = degrees of freedom; CFI = Comparative Fit Index; TLI = Tucker-Lewis Index; RMSEA = Root Mean Square Error of Approximation; CI = Confidence Interval; WRMR = Weighted Root Mean Square Residual.

In addition, we calculated the standardized factor loadings for this solution that were all significant and higher than .30 (see Table 2).

**Table 2:** Factor loadings of the general factor and the specific factors of the bifactor model.

Item	Factor Loading	Item	Factor Loading	Item	Factor Loading	Item	Factor Loading
**General Factor**							
SE1	0.70	AU10	0.51	IL8	0.43	OP11	0.50
SE2	0.62	AU11	0.43	IL9	0.46	ST1	0.39
SE3	0.65	AU12	0.44	IL10	0.35	ST2	0.37
SE4	0.70	AU13	0.45	AM1	0.48	ST3	0.31
SE5	0.67	AU14	0.39	AM2	0.48	ST4	0.40
SE6	0.78	IN1	0.42	AM3	0.49	ST5	0.52
SE7	0.59	IN2	0.35	AM4	0.35	ST6	0.54
SE8	0.54	IN3	0.61	AM5	0.39	ST7	0.58
SE9	0.53	IN4	0.57	AM6	0.36	ST8	0.56
SE10	0.57	IN5	0.30	AM7	0.38	ST9	0.34
SE11	0.72	IN6	0.32	AM8	0.54	ST10	0.46
SE12	0.39	IN7	0.41	AM9	0.53	ST11	0.48
SE13	0.38	IN8	0.43	AM10	0.52	RT1	0.41
SE14	0.42	IN9	0.49	AM11	0.51	RT2	0.42
SE15	0.40	IN10	0.53	AM12	0.56	RT3	0.38
SE16	0.61	IN11	0.39	AM13	0.50	RT4	0.32
SE17	0.31	IN12	0.38	AM14	0.38	RT5	0.35
SE18	0.42	IN13	0.37	OP1	0.39	RT6	0.31
AU1	0.49	IN14	0.36	OP2	0.30	RT7	0.48
AU2	0.58	IN15	0.49	OP3	0.45	RT8	0.37
AU3	0.53	IL1	0.58	OP4	0.56	RT9	0.36
AU4	0.58	IL2	0.50	OP5	0.47	RT10	0.31
AU5	0.60	IL3	0.43	OP6	0.49	RT11	0.47
AU6	0.61	IL4	0.48	OP7	0.35	RT12	0.46
AU7	0.63	IL5	0.46	OP8	0.36	RT13	0.52
AU8	0.68	IL6	0.47	OP9	0.47	RT14	0.59
AU9	0.70	IL7	0.42	OP10	0.52		
**Group Factors**							
**SE**	**AU**	**IN**	**IL**	**AM**	**OP**	**ST**	**RT**
0.14	0.19	0.25	0.12	0.16	0.18	-0.10	0.41
0.28	0.08	0.35	0.05	0.15	0.16	0.25	0.15
0.30	0.16	0.40	0.42	0.12	0.25	0.16	0.12
0.15	0.35	0.41	0.26	0.28	0.26	0.19	0.13
-0.14	0.15	0.43	0.25	0.11	0.28	0.29	0.11
0.40	0.19	0.34	0.16	0.41	0.32	0.35	0.28
0.16	0.21	0.09	0.41	0.43	0.37	0.38	0.32
0.15	0.25	0.10	0.42	0.28	0.36	0.36	0.41
0.17	0.34	0.15	0.33	0.35	0.39	0.30	0.39
0.19	0.33	0.25	0.16	0.50	0.41	0.41	0.45
0.20	0.41	0.19		0.41	0.45	0.29	0.48
0.31	0.12	0.15		0.38			0.36
0.29	0.25	0.16		0.15			0.16
0.41	0.31	0.19		0.25			0.15
0.29		0.15					
0.37							
0.30							
0.36							

Note. SE, self-efficacy; AU, autonomy; IN, innovativeness; IL, internal locus of control; AM, achievement motivation; OP, optimism; ST, stress tolerance; RT, risk-taking.

### Analysis of the measurement invariance of the BEPE

We then studied factorial equivalence of the bifactor model across a) gender (men versus women), and b) age (younger -12 to 15 years old- vs older adolescents -15 to 19 years old-). To examine MI across age, the sample was divided into two different subgroups: 12–15 years old (n = 835), and 16–19 years old (n = 270), attending to the initial and final stages of adolescence [40].

Firstly, we tested the fit of the bifactor model in each group separately. Secondly, configural and scalar invariance were examined (see Table 3). As seen in Table 3, differences in CFI (ΔCFI) below .01 between the configural and scalar were found across gender and age. Therefore, the hypothesis of MI was confirmed both by gender and age.

**Table 3:** Goodness-of-fit indices for measurement invariance of the BEPE-A (Bifactor model) across gender and age.

	χ^2^	*Df*	CFI	TLI	RMSEA (90% C.I)	WRMR	ΔCFI
**Gender (Men Vs Women)**							
Men (*n* = 528)	587.98	148	.91	.91	.05 (.05-.06)	1.30	
Women (*n* = 577)	566.75	148	.92	.92	.05 (.05-.06)	1.28	
Configural Invariance	576.99	315	.92	.91	.05 (.05-.06)	1.32	
Strong factorial invariance	949.54	357	.91	.90	.05 (.05-.06)	1.84	-.01
**Age (Younger Vs Older Adolescents)**							
Younger 12–15 (*n* = 835)	553.64	148	.90	.90	.06 (.05-.06)	1.23	
Older 16–19 (*n* = 270)	510.44	148	.91	.92	.05 (.05-.06)	1.18	
Configural Invariance	515.69	315	.91	.91	.06 (.05-.06)	1.67	
Scalar invariance	589.21	357	.91	.92	.06 (05-.06)	1.86	-.01

*Note*. χ^2^ = Chi square; *df* = degrees of freedom; CFI = Comparative Fit Index; TLI = Tucker-Lewis Index; RMSEA = Root Mean Square Error of Approximation; C.I = Confidence Interval; WRMR = Weighted Root Mean Square Residual. ΔCFI = Change in Confirmatory Fit Index.

### Latent mean comparisons

The study of the latent mean differences revealed that men scored higher than women in entrepreneurial initiative (.08; *p* < .01; *d* = 0.15), optimism (.13; *p* < .01; *d* = 0.21), and locus of control (.12; *p* < .01; *d* = .22). With regards to age, older adolescents scored higher than younger adolescents in stress tolerance (.11; *p* < .01; *d* = .20). Considering the effect size all the found with regard to gender and age were small with d ≤ .30.

### Descriptive statistics for the BEPE-A dimensions by gender and age

Once the bifactor model was retained as the model with a better fit considering both the fit indices and the factor loadings, descriptive statistics and internal consistency for this model were calculated (see Table 4) for the different factors and the general factor. Internal consistency values for the BEPE-A dimensions ranged between .79 (Innovativeness) and .83 (Autonomy) estimated using McDonald’s Omega.

**Table 4:** Descriptive statistics and internal consistency scores for the BEPE subscales and the general factor.

	Group Factors		General Factor	
	Mean (SD)	Omega	Mean (SD)	Omega
Self-efficacy	36.28 (4.4)	.81	35.40 (4.59)	.82
Autonomy	37.42 (4.9)	.83		
Innovativeness	39.02 (5.1)	.79		
Internal locus of control	38.76 (5.03	.78		
Achievement motivation	39.18 (4.95)	.82		
Optimism	38.45 (5.21)	.83		
Stress tolerance	33.23 (5.16)	.77		
Risk Taking	37.19 (5.24)	.80		

*Note*. SD = Standard Deviation.

### Evidences of relation with other measures

Correlations between the BEPE dimensions and the EASS dimensions were statistically significant (see Table 5). The Pearson’s correlations ranged between Self-efficacy of the BEPE-A and Autonomy of the EASS (.10) and Autonomy of the BEPE-A and Autonomy of the EASS (.79). As it was hypothesized, the EASS dimensions were statistically significant when associated with the BEPE-A dimensions.

**Table 5:** Pearson’s correlations between the BEPE-A dimensions and total score and the EASS subscales.

	EASS						
BEPE-A	Pr	PE	EM	IN	AU	RT	Total
Self-efficacy	.43	.28	.33	.26	.10	.39	.49
Autonomy	.39	.31	.24	.38	.79	.43	.38
Innovativeness	.42	.30	.23	.74	.36	.51	.36
Internal locus of control	.38	.29	.30	.52	.44	.47	.43
Achievement Motivation	.41	.19	.29	.37	.34	.49	.49
Optimism	.36	.22	.24	.31	.33	.38	.41
Stress tolerance	.37	.27	.33	.30	.32	.41	.38
Risk Taking	.45	.25	.27	.37	.40	.76	.34

*Note*. All the correlations were statistically significant *p* ≤ .01; Pr = Proactivity; PE = Professional Ethic; EM = Empathy; IN = Innovativeness; AU = Autonomy, RT = Risk Taking.

## Discussion

At this moment, and considering the continuous and sudden changes in the market, the ability to innovate and detect new possibilities to grow is a priority. Thus, it is relevant to detect and then promote entrepreneurship at early ages. To this aim, instruments with evidences of validity and reliability are needed. The main goal of the present study was to analyze entrepreneurship in adolescents at school and gather evidences of validity and reliability of the BEPE-A, a pool of items based on computerized adaptive assessment shorter than the previous BEPE form. To the best of our knowledge, this study constitutes the first attempt to reduce the amount of a previously validated measurement instrument like the BEPE, preserving the 8 dimensions. Analyzing and studying aspects related to entrepreneurship in adolescents is relevant and could allow implementation of early strategies that improve these aspects during a developmentally important stage such as adolescence. For this purpose, shorter measurement tools with adequate psychometric properties are required, as they improve satisfaction and are less time consuming [46].

The results found in the present study from the EFA revealed that each of the dimensions of BEPE-A was basically unidimensional. This is congruent with previous studies using both the BEPE [8] and the BEPE-A [18]. In addition, the study of the CFA revealed that a bifactor structure with 8 separate dimensions and a general factor of enterprising, better explained the BEPE-A structure. The results of both the EFA and the CFA support the idea of the BEPE-A as an instrument that provides valid measures of 8 different domains of enterprising personality. It also confirms that these dimensions, although measuring different domains, share a common structure related to the entrepreneurial personality. The original idea of the BEPE was precisely configuring an enterprising profile of personality [3,8].

With regards to the study of internal consistency of the scores, the BEPE-A scores showed adequate levels of reliability by means of McDonald’s Omega. Previous studies have shown similar results with the BEPE and the BEPE-A [9,18]. Thus, it is possible to confirm that the BEPE-A has evidences of content validity and internal consistency of the scores.

Moreover, results support the hypothesis of MI of the bifactor model of the BEPE-A both by gender and age. To date, and to the best of our knowledge, no previous studies have analyzed the MI of the BEPE or the BEPE-A by these relevant variables. The differential item functioning, a somehow similar technique, revealed that some items of the BEPE showed a different functioning attending to these variables [8] and the BEPE-A [18]. The different and significant biopsychosocial changes that occur during adolescence impact in a different way depending on gender and age [40]. Therefore, screening and psychological assessment of aspects like entrepreneurship should address the possibility that different theoretical constructs may be understood differently in response to variables like gender or age. If MI does not hold, the results based on comparisons across these variables may be unfounded and therefore not valid [36]. MI has to be demonstrated for a meaningful comparison of measuring constructs across groups. Thus, the use of MI or DIF regarding specific instruments aimed to assess enterprising personality is still needed. Therefore, the present study contributes valuable information allowing the comparability of entrepreneurial scores across relevant variables such as gender or age.

The study of latent mean differences revealed that, on average, men showed more entrepreneurial initiative, optimism, and locus of control than women, whereas younger adolescents revealed less stress tolerance than older adolescents. Nonetheless, the effect size for the differences was small [42]. Previous studies have shown that women had more emotional problems and somehow less refined self-regulation than men [47,48]. Similarly, other studies have also shown that women are less likely to engage in risk-taking behaviours, being more conservative [19,20] but also with small effect sizes. In addition, previous studies [49] revealed that younger populations of students had more ambition while adults showed more self-assurance and self-control. These results seem to be congruent with data found in the present work. However, it is worth mentioning that other studies have revealed that older participants had higher levels of entrepreneurial activity [21]. Also, maturation of executive functions during adolescence [50] may be the explanation for a higher tolerance to stress and frustration in older adolescents. The study of enterprising personality may allow us to detect and implement educational strategies for women and men as well as for younger and older adolescents that can contribute to an optimal develop of these transcendent skills. Training in entrepreneurial activity should be considered as a key aspect when designing educational policies with the aim to further develop entrepreneurship of women and men, as well as younger and older adolescents.

Finally, the BEPE-A revealed adequate evidences of relationship with other variables. The study of the Pearson’s correlations showed a statistically significant and positive correlation between all the BEPE-A subscales and the EASS subscales. These results support the validity of the BEPE-A (Spanish version) with other external variables and are consistent with previous studies, where the BEPE subscales were found to correlate, for instance with the META subscales [10].

The present study should be seen in light of the following limitations. First, the BEPE-A, was applied in regular basis and all the participants received the total pool of items. The cross-sectional nature of the study prevents establishing cause-effect relationships. Also, there are an inherent problems in the use of self-reports questionnaires. Also, we studied adolescents at school in a particular region of the north of Spain. This, precludes the generalization of the results to the rest of the Spanish territory.

Notwithstanding these limitations, the results found in the present study have relevant implications. The study of evidences of an instrument such as the BEPE allow to generate and assess profiles of enterprising personality. In this sense, the BEPE-A is a shorter instrument, in contrast to the BEPE that has shown adequate evidences of validity and internal consistency of the scores. Considering the global socioeconomical situation and the sudden changes that characterize society and economy nowadays, it seems reasonable to think that early detection and promotion of entrepreneurial personality is not only adequate but necessary. Bearing this in mind, having a valid and reliable instrument that allows measuring enterprising personality becomes not only valuable but necessary. Therefore, more studies should continue to analyzing the psychometric properties of instruments like the BEPE and the BEPE-A. In addition, considering the fact that one of the limitations of the BEPE-A is that, although shorter than its predecessors, it is still time-demanding for use in school settings, and it would be beneficial to study new versions with a reduce number of items per dimension.
